# Hepatitis C Virus Clearance with Glucose Improvement and Factors Affecting the Glucose Control in Chronic Hepatitis C Patients

**DOI:** 10.1038/s41598-020-58786-x

**Published:** 2020-02-06

**Authors:** Man Yuan, Juan Zhou, Lingyao Du, Libo Yan, Hong Tang

**Affiliations:** 1 Center of Infectious Diseases, West China Hospital of Sichuan University, Chengdu, China; 2 Division of Infectious Diseases, State Key Laboratory of Biotherapy and Center of Infectious Diseases, West China Hospital, Sichuan University, Chengdu, China; 3 Department of Laboratory Medicine, West China Hospital, Sichuan University, Chengdu, China

**Keywords:** Hepatitis C, Hepatitis C

## Abstract

The study aimed to investigate whether the glucose level improves and what factors affect the improvement in glucose control after the eradication of hepatitis C virus (HCV). A total of 1090 patients with HCV infections were enrolled, among which 278 (25.5%) patients were diagnosed with prediabetes, and 89 (8.16%) patients were diagnosed with diabetes. In the cohort, 990 patients belonged to sustained virological response (SVR) group and 100 belonged to non-SVR group. Decreases in the fasting plasma glucose (FPG) level were found in the SVR group but not in the non-SVR group (p < 0.001; p = 0.267). In the SVR group, subjects with baseline FPG ≥ 5.6 mmol/L were further stratified into glycometabolism-improved (N = 182) and unimproved (N = 150) groups according to their FPG after viral eradication. Multivariate analysis showed that older age, higher baseline HCV RNA, glucose, total bilirubin and alanine aminotransferase levels were independent risk factors for insufficient glucose improvement. In conclusion, patients with HCV infection had a higher prevalence of abnormal glycometabolism. It could be improved after viral eradication, indicating that HCV may influence glycometabolism. Moreover, Age, baseline HCV RNA, glucose, total bilirubin and alanine aminotransferase levels were impact factor for glycometabolism improvement after viral eradication.

## Introduction

Hepatitis C virus (HCV) infection is a major health burden and one of the most prevalent communicable diseases. It is estimated that at least 150–170 million people worldwide are chronically infected, and those with persistent infections may develop cirrhosis and hepatocellular carcinoma and die due to liver-related causes^1^. On the other hand, diabetes mellitus is one of the most prevalent non-communicable diseases worldwide, and more than 420 million people are currently suffering it^2^. Moreover, people with impaired fasting glucose (IFG) have been referred to as having prediabetes, which indicates a higher risk for future diabetes development. As a result, the global prevalence of diabetes is expected to reach 642 million individuals by 2040, which will cause a direct and indirect social burden^3^. Epidemiologic studies supporting the association between HCV infection and type 2 diabetes mellitus (T2DM) have been available for over 20 years^4–7^. HCV infection increases the risk of T2DM^8^. A large-scale study showed that the prevalence of positive anti-HCV antibodies was 13.7% in T2DM patients, while it was only 4.9% in volunteer blood donors without diabetes^9^. In addition, in HCV-infected people, the prediabetes prevalence rate was reported to be as high as 37%^10^. Currently, T2DM is regarded as an extrahepatic manifestation of HCV infection to some degree^11^. Insulin resistance (IR) is a critical cause of T2DM. Although the specific mechanisms are not fully elucidated, evidence suggests that HCV can interfere with the insulin signaling pathway^12,13^. HCV can activate inhibitors of insulin signaling by degrading insulin receptor substrate-1 (IRS-1), upregulating cytokine signaling-3 (SOCS-3), activating mammalian target of rapamycin (mTOR) and c-Jun N-terminal kinase (JNK)^14–17^. HCV can also increase endoplasmic reticulum (ER) stress to upregulate the expression of the protein phosphatase 2A (PP2A), inhibiting gluconeogenesis^18^. Owing to the association between HCV and T2DM, HCV eradication may lead to an improvement in IFG/T2DM. However, there are conflicting data regarding the improvement in IFG/T2DM in patients who previously had HCV infections and achieved a sustained virologic response (SVR). Some studies showed a decreased rate of glucose abnormalities in such cohorts^19–22^, whereas a few studies failed to demonstrate a statistically significant decrease in the incidence of glucose abnormalities in these patients^23,24^. In this study, we aimed to investigate whether the glucose level improved and what factors affected the glucose improvement in patients with previous HCV infections who achieved an SVR.

## Materials and Methods

### Study subjects

In this retrospective study, we reviewed the medical records of 1090 Chinese Han patients with previous HCV infections aged over 18 who received pegylated interferon-α and ribavirin (PR) treatment at West China Hospital of Sichuan University from January 2008 to January 2017. Written informed consent was obtained from all subjects, and the study protocol was approved by the Ethics Committee of the West China Hospital of Sichuan University (Chengdu, Sichuan, China) in accordance with the ethical guidelines of the 1975 Declaration of Helsiniki. Subjects with other clinical liver diseases, such as alcoholic liver disease, autoimmune hepatitis or toxic hepatitis, and hepatocellular carcinoma, and with human immunodeficiency virus or hepatitis B virus coinfection were excluded.

### Definitions of treatment responses and glucose improvement

SVR was defined as undetectable HCV RNA levels at 24 weeks after the end of treatment (EOT). Fasting plasma glucose (FPG) levels between 100–125 mg/dL (5.6–6.9 mmol/L) were defined as prediabetes and FPG ≥ 126 mg/dL (7.0 mmol/L) was defined as diabetes^3^. In the SVR group, subjects with baseline FPG ≥ 5.6 mmol/L were stratified into the glucose-improved and unimproved groups according to whether they achieved a significant decline in FPG at 24 weeks after the EOT. A significant decline in FPG was defined as patients with a prediabetes status who achieved an FPG < 5.6 mmol/L or patients with a diabetes status who achieved an FPG < 7 mmol/L.

### Laboratory assays

All parameters were measured at the central lab of West China Hospital of Sichuan University. Anti-HCV antibodies were detected by a Modular E170 analyzer (Roche Diagnostics, Munich, Bavaria, Germany). The HCV genotype was identified with Sanger sequencing (Thermo Fisher Scientific, Waltham, MA, USA). The lower limit of detection for serum HCV RNA loads was 20 IU/mL, as measured by a real-time polymerase chain reaction assay (Lightcycler-480; Roche, Basel, Switzerland). Biochemical indicators, such as total bilirubin (TBiL), alanine aminotransferase (ALT), aspartate aminotransferase (AST), albumin (ALB), alkaline phosphatase (ALP), and gamma glutamyltranspeptidase (GGT) were tested by a colorimetric method (Modular EVO; Roche, Basel, Switzerland). Hemoglobin (Hb), white blood cells (WBCs), platelets (PLTs) were tested by a Sysmex XS-2000i autoanalyzer (Sysmex Corporation, Kobe, Hyogo, Japan). FPG was measured by an automatic biochemical analyzer (Modular DDP, Roche, Bruchsal, Germany). HbA1C (hemoglobin A1C) was measured on a Tosoh G7 in the standard mode (Tosoh Corporation, Tokyo, Japan). The baseline and post-treatment FPG, HbA1C, TBiL, ALT, AST, ALB, ALP, GGT, Hb, WBCs and PLTs were collected within two weeks before treatment and at 24 weeks after the EOT. The AST to platelet ratio index (APRI) ((AST/ULN) × 100/(PLT(10^9^/L)) and Fibrosis-4 (FIB-4)-index (age × AST/(PLT(10^9^/L) × ALT ^1/2^)) were used to assess the liver stiffness.

### Statistical analyses

All statistical analyses were performed using SPSS version 22.0 (SPSS, Inc., Chicago, IL, USA). Continuous variables without a normal distribution are expressed as the median and inter-quartile range (IQR). Categorical variables are expressed as frequencies and percentages. The χ^2^ test or Fisher’s exact probability test was used to examine categorical variables such as sex, age and HCV genotype. The Mann-Whitney U test was used to examine continuous variables in the SVR and non-SVR groups, and the improved and unimproved groups. The Wilcoxon rank-sum test was used to examine the differences in pre-treatment and post-treatment profiles in the SVR and non-SVR groups. To evaluate the impact of baseline profiles on the improvement in glucose in patients with prediabetes/diabetes in the SVR group, we categorized all continuous variables (except APRI and FIB-4) into four groups based on their medians and inter-quartile ranges. Associations between predictor variables and glucose improvement were determined by the odds ratio (OR) and 95% confidence interval (CI), which were calculated using binary logistic regression. The propensity score matching (PSM) method was performed to balance the baseline characteristics of the SVR and non-SVR groups. A two-sided P-value < 0.05 was considered statistically significant.

## Results

### The selected study cohort and the baseline characteristics of patients with HCV infections before treatment

**Figure 1:**
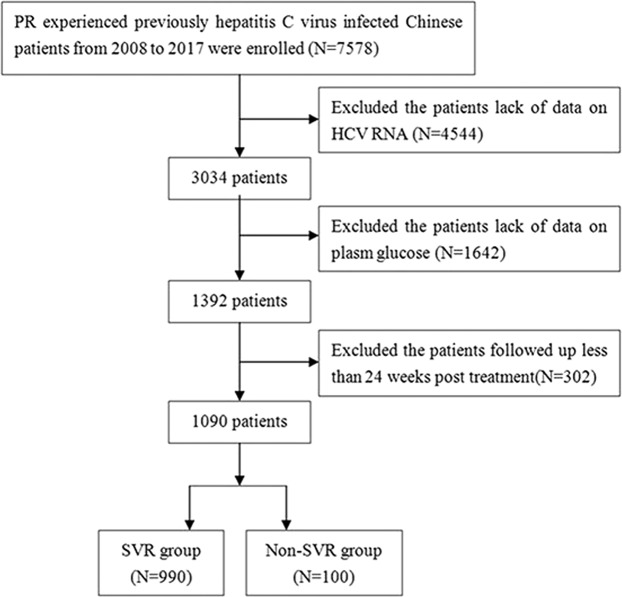
Flow chart of study population selection. PR, pegylated interferon-α and ribavirin; HCV RNA, hepatitis C virus ribonucleic acid; SVR, sustained virologic response; non-SVR, non sustained virologic response.

A diagram of study population selection is shown in Fig. 1. A total of 1090 patients with HCV infections were enrolled in the final cohort for sequential analysis. The baseline clinical characteristics of these 1090 Chronic Hepatitis C (CHC) patients are listed in Table 1. Their median age was 48 (IQR 40–57) years old. The distribution of HCV genotypes was as follows: 65.9% (N = 718) belonged to genotype 1, 4.6% (N = 50) belonged to genotype 2, 7.7% belonged to genotype 3 (N = 84) and the remaining 21.8% belonged to other and unknown genotypes (N = 238). Additionally, a total of 278 (25.5%) patients were diagnosed with prediabetes, and 89 (8.16%) patients were diagnosed with diabetes. Among the 89 T2DM patients, thirty-five (39.3%) patients received antidiabetic medications, and 54 (60.7%) received lifestyle treatment alone (Supplementary Table S1). Among the 1090 patients, 990 had an SVR, and the remaining 100 had a non-SVR. The ALB, Hb and WBCs levels in the SVR group were significantly higher than those in the non-SVR group. There were no significant differences in the distribution of sex, age, genotype, HCV RNA, FPG,  diabetes status, TBiL, ALT, AST, ALP, GGT, PLTs, APRI and FIB-4 between the SVR and non-SVR groups.

**Table 1:** Baseline characteristics of the CHC patients before anti-HCV treatment.

Parameters	All patients (N = 1090)	SVR(+) (N = 990)	SVR(−) (N = 100)	*P*-value^a^ SVR(+) vs. SVR(−)
Male	490(45%)	44(44.9%)	45(45%)	0.992
Age, years	48(40–57)	48(40–57)	49(40–56)	0.993
Genotype				
1	718(65.9%)	650(65.7%)	68(68%)	0.610
2	50(4.6%)	48(4.8%)	2(2%)	
3	84(7.7%)	77(7.8%)	7(7%)	
Others and unknown	238(21.8%)	215(21.7%)	23(23%)	
HCV-RNA, log IU/mL	6.06(5.10–6.55)	6.05(5.11–6.54)	6.20(4.64–6.66)	0.419
FPG, mmol/L	5.31(4.94–5.85)	5.31(4.93–5.85)	5.34(5.02–5.83)	0.364
Prediabetes	278(25.5%)	250(25.2%)	28(28%)	0.537
Diabetes	89(8.16%)	82(8.28%)	7(7%)	
TBiL, μmol/L	15.2(11.5–20.6)	15.2(11.5–20.5)	15.6(10.92–21.4)	0.822
ALT, IU/L	49(26–98)	49(26–99)	47(27–84)	0.594
AST, IU/L	45(30–78)	46(46–79)	42(30–76.75)	0.867
ALB, g/L	44(41.1–46.5)	44.1(41.2–46.6)	43.25(40.80–45.47)	0.038^b^
ALP, IU/L	82(68–106)	82(67–106)	86.50(71–105.75)	0.218
GGT, IU/L	34.50(19–76)	34(19–73.25)	39.5(39.5–81.75)	0.301
Hb, g/L	136(123–150)	137(123.75–150)	131.5(111.5–142)	0.003^b^
PLTs, 10^9^/L	121(85–165)	123(86–166)	111(72.25–156.50)	0.147
WBCs, 10^9^/L	5.02(3.91–6.35)	5.07(3.98–6.42)	4.34(4.34–5.655)	<0.001^b^
APRI	0.99(0.54–1.99)	0.98(0.54–1.98)	1.17(0.55–2.21)	0.522
FIB-4	2.63(1.49–5.09)	2.61(1.47–5.08)	2.69(1.73–5.28)	0.315

CHC, chronic hepatitis C; HCV, Hepatitis C virus; SVR, sustained virologic response; HCV RNA, hepatitis C virus ribonucleic acid; FPG, fasting plasma glucose; TBiL, total bilirubin; ALT, alanine aminotransferase; AST, aspartate aminotransferase; ALB, albumin; ALP, alkaline phosphatase; GGT, gamma glutamyltranspeptidase; Hb, hemoglobin; PLTs, platelets; WBCs, white blood cells; APRI, aspartate aminotransferase to platelet ratio index; FIB-4, Fibrosis-4. ^a^The χ^2^ test or Fisher’s exact probability test was used to examine the categorical variables such as sex, age genotype and diabetes status, and the Mann-Whitney U test was used to examine the continuous variables between SVR (+) and SVR (−) group. ^b^Values were statistically significant at *P* < 0.05.

### Changes in clinical parameters between the SVR and non-SVR groups

The changes in various clinical parameters before and after treatment are shown in Table 2. After treatment, the serum levels of TBiL, AST, GGT, and APRI decreased, whereas the levels of ALB and PLT increased both in the SVR and non-SVR groups. These results suggested that the eradication of HCV improved liver function. Moreover, decreases in the FPG level were found in the SVR group but not in the non-SVR group (P < 0.001; p = 0.267), indicating that a correlation may exist between improvements in FPG and the eradication of HCV. As shown in Supplementary Table S2, in the T2DM CHC patients with an SVR, the HbA1C level also decreased after viral eradication (P = 0.002).

**Table 2:** Comparison of baseline and post-treatment variables in the CHC patients with and without an SVR.

Parameters	SVR(+) (N = 990)		*P*-value^a^	SVR(−) (N = 100)		*P*-value^a^
	Pre-treatment	Post-treatment		Pre-treatment	Post-treatment	
HCV-RNA, log IU/mL	6.05(5.11–6.54)	0	<0.001^b^	6.20(4.64–6.66)	4.71(3.28–6.11)	<0.001^b^
FPG, mmol/L	5.31(4.93–5.85)	5.13(4.76–5.59)	<0.001^b^	5.34(5.02–5.83)	5.42(4.91–5.99)	0.267
TBiL, μmol/L	15.2(11.5–20.5)	13.7(10.2–18.2)	<0.001^b^	15.6(10.92–21.4)	12.75(9.42–19.45)	0.002^b^
ALT, IU/L	49(26–99)	21(15–32)	<0.001^b^	47(27–84)	35.50(19.25–68)	0.157
AST, IU/L	46(46–79)	27(21–35)	<0.001^b^	42(30–76.75)	34(26.25–58)	0.040^b^
ALB, g/L	44.1(41.2–46.6)	45.9(43.175–48)	<0.001^b^	43.25(40.80–45.47)	45.35(42.72–47.3)	<0.001^b^
ALP, IU/L	82(67–106)	79(65–98)	<0.001^b^	86.50(71–105.75)	86(72–100.75)	0.443
GGT, IU/L	34(19–73.25)	20(13–37)	<0.001^b^	39.5(39.5–81.75)	31(20–56.75)	0.004^b^
Hb, g/L	137(123.75–150)	136(121–149)	0.002^b^	131.5(111.5–142)	130.5(111.25–147.75)	0.835
PLTs, 10^9^/L	123(86–166)	131(91–176)	<0.001^b^	111(72.25–156.50)	120(86–172)	0.022^b^
WBCs, 10^9^/L	5.07(3.98–6.42)	5.2(4.07–6.40)	0.083	4.34(4.34–5.65)	4.53(3.43–6.03)	0.115
APRI	0.98(0.54–1.98)	0.52(0.33–0.89)	<0.001^b^	1.17(0.55–2.21)	0.71(0.48–1.42)	0.042^b^
FIB-4	2.61(1.47–5.08)	2.30(1.33–3.83)	<0.001^b^	2.69(1.73–5.28)	2.43(1.49–4.25)	0.081

CHC, chronic hepatitis C; SVR, sustained virologic response; HCV RNA, hepatitis C virus ribonucleic acid; FPG, fasting plasma glucose; TBiL, total bilirubin; ALT, alanine aminotransferase; AST, aspartate aminotransferase; ALB, albumin; ALP, alkaline phosphatase; GGT, gamma glutamyltranspeptidase; Hb, hemoglobin; PLTs, platelets; WBCs, white blood cells; APRI, aspartate aminotransferase to platelet ratio index; FIB-4, Fibrosis-4. ^a^The Wilcoxon rank-sum test was used to examine the differences between pre-treatment and post-treatment profiles in SVR (+) and SVR (−) group, respectively. ^b^Values were statistically significant at P < 0.05.

### Changes in clinical parameters in the SVR and non-SVR groups after propensity score matching

We used PSM to normalize the baseline characteristics between the SVR and non-SVR groups. The normalized characteristics included “age, genotype, HCV-RNA, FPG, TBiL, ALT, AST, ALB, ALP, GGT, Hb, PLTs, WBCs, APRI and FIB-4”. After PSM, we enrolled 99 patients in the SVR group and 99 patients in the non-SVR group. The baseline clinical characteristics of the 198 HCV patients with HCV infections are listed in Table 3. PSM guaranteed that there were no significant differences in the distribution of sex, age, genotype, HCV RNA, FPG, TBiL, ALT, AST, ALP, GGT, PLTs, APRI and FIB-4 between the SVR and non-SVR groups at baseline (all P > 0.05). The changes in these clinical parameters before and after treatment were further analyzed and are shown in Table 4. We found decreases in the FPG level in patients with SVR but not in the non-SVR group after PSM (P = 0.027; p = 0.723). This result further demonstrated that the clearance of HCV may improve glucose metabolism.

**Table 3:** Baseline characteristics of the CHC patients before anti-HCV treatment after propensity score matching.

Parameters	SVR (+) (N = 99)	SVR (−) (N = 99)	*P* - value^a^ SVR(+) vs. SVR(−)
Male	46(46.5%)	44(44.4%)	0.775
Age, years	47(41–57)	49(40–56)	0.860
Genotype			
1	59(59.6%)	67(67.6%)	0.134
2	7(7%)	2(2%)	
3	12(12.1%)	7(7%)	
Others and unknown	21(21.2%)	23(23.2%)	
HCV-RNA, log IU/mL	5.84(4.66–6.66)	6.19(4.64–6.64)	0.456
FPG, mmol/L	5.35(4.99–5.98)	5.33(5.02–5.83)	0.900
Prediabetes	21(21.2%)	27(27.2%)	0.285
Diabetes	10(10.1%)	7(7.1%)	
TBiL, umol/L	15.1(11.2–21.2)	15.6(10.9–21.4)	0.948
ALT, IU/L	54(29–97)	47(27–84)	0.432
AST, IU/L	42(31–74)	42(30–76)	0.934
ALB, g/L	44.4(41.3–46.8)	43.2(40.80–45.4)	0.045^b^
ALP, IU/L	83(68–106)	86(71–105)	0.541
GGT, IU/L	36(24–67)	38(20–78)	0.954
Hb, g/L	142(125–153)	131(111–142)	0.001^b^
PLTs, 10^9^/L	128(96–172)	111(72–157)	0.094
WBCs, 10^9^/L	5.24(4.14–6.9)	4.35(3.46–5.67)	0.001^b^
APRI	0.91(0.55–1.63)	1.17(0.55–2.21)	0.326
FIB-4	2.33(1.52–4.13)	2.69(1.73–5.33)	0.181

CHC, chronic hepatitis C; HCV, Hepatitis C virus; SVR, sustained virologic response; HCV RNA, hepatitis C virus ribonucleic acid; FPG, fasting plasma glucose; TBiL, total bilirubin; ALT, alanine aminotransferase; AST, aspartate aminotransferase; ALB, albumin; ALP, alkaline phosphatase; GGT, gamma glutamyltranspeptidase; Hb, hemoglobin; PLTs, platelets; WBCs, white blood cells; APRI, aspartate aminotransferase to platelet ratio index; FIB-4, Fibrosis-4. ^a^The χ^2^ test or Fisher’s exact probability test was used to examine the categorical variables such as sex, age, genotype and diabetes status, and the Mann-Whitney U test was used to examine the continuous variables between SVR (+) and SVR (−) group. ^b^Values were statistically significant at P < 0.05.

**Table 4:** Comparison of baseline and post-treatment variables in the CHC patients with and without an SVR after propensity score matching.

Parameters	SVR(+) (N = 99)		*P*-value^a^	SVR (−) (N = 99)		*P*-value^a^
	Pre-treatment	Post-treatment		Pre-treatment	Post-treatment	
HCV-RNA, log IU/mL	5.84(4.66–6.66)	0	<0.001^b^	6.19(4.64–6.64)	4.69(3.23–6.06)	<0.001^b^
FPG, mmol/L	5.35(4.99–5.98)	5.14(4.81–5.62)	0.027^b^	5.33(5.02–5.83)	5.42(4.91–5.96)	0.723
TBiL, umol/L	15.1(11.2–21.2)	13.60(9.6–18.1)	0.055	15.6(10.9–21.4)	12.7(9.4–19)	0.037^b^
ALT, IU/L	54(29–97)	20 (14–31)	<0.001^b^	47(27–84)	35(19–68)	0.049^b^
AST, IU/L	42(31–74)	25(21–33)	<0.001^b^	42(30–76)	34(26–58)	0.023^b^
ALB, g/L	44.4(41.3–46.8)	45.8(43.4–47.6)	0.004^b^	43.2(40.80–45.4)	45.3(42.7–47.3)	0.001^b^
ALP, IU/L	83(68–106)	78(62–92)	0.050	86(71–105)	86(72–100)	0.579
GGT, IU/L	36(24–67)	20(14–35)	<0.001^b^	38(20–78)	31(20–56)	0.105
Hb, g/L	142(125–153)	141(117–151)	0.407	131(111–142)	130(111–148)	0.958
PLTs, 10^9^/L	128(96–172)	138(103–174)	0.356	111(72–157)	120(86–172)	0.273
WBCs, 10^9^/L	5.24(4.14–6.9)	5.75(3.86–7.3)	0.443	4.35(3.46–5.67)	4.49(3.43–6)	0.310
APRI	0.91(0.55–1.63)	0.49(0.33–0.76)	<0.001^b^	1.17(0.55–2.21)	0.71(0.48–1.37)	0.020^b^
FIB-4	2.33(1.52–4.13)	2.13(1.38–3.24)	0.126	2.69(1.73–5.33)	2.43(1.49–4.07)	0.244

CHC, chronic hepatitis C; SVR, sustained virologic response; HCV RNA, hepatitis C virus ribonucleic acid; FPG, fasting plasma glucose; TBiL, total bilirubin; ALT, alanine aminotransferase; AST, aspartate aminotransferase; ALB, albumin; ALP, alkaline phosphatase; GGT, gamma glutamyltranspeptidase; Hb, hemoglobin; PLTs, platelets; WBCs, white blood cells; APRI, aspartate aminotransferase to platelet ratio index; FIB-4, Fibrosis-4. ^a^The Wilcoxon rank-sum test was used to examine the differences between pre-treatment and post-treatment profiles in SVR (+) and SVR (−) group, respectively. ^b^Values were statistically significant at P < 0.05.

### Baseline clinical characteristics of SVR patients with improved glucose levels and unimproved glucose levels

We selected 332 patients with baseline FPG levels ≥ 5.6 from the SVR group (Table 5). Among these patients, 182 patients had an improvement in glucose level after SVR was achieved, while 150 patients did not. The median age of patients in the improved group (49 (IQR 41–59.25)) was lower than that in the unimproved group (54 (IQR 46.75–62)). Moreover, lower ALP, APRI and FIB-4 levels were significantly more common in the glucose improved group than the unimproved group. This result implied that the improvement in plasma glucose occurred along with improvements in some liver-related parameters after the eradication of HCV.

**Table 5:** Baseline characteristics of the CHC patients with an SVR with baseline glucose levels ≥ 5.6.

Parameters	All (N = 332)	Improved (N = 182)	Unimproved (N = 150)	*P* - value^a^
Male	173(52.1%)	96(52.7%)	77(51.3%)	0.797
Age, years	51(44–61)	49(41–59.25)	54(46.75–62)	0.004^b^
Genotype				
1	217(65.4%)	116(63.7%)	101(67.3%)	0.604
2	11(3.3%)	5(2.7%)	6(4%)	
3	27(8.1%)	14(7.7%)	13(8.7%)	
Others and unknown	77(23.2%)	47(25.8%)	30(20%)	
HCV-RNA, log IU/mL	6.07(5.17–6.55)	6.06(4.95–6.56)	6.08(5.31–6.56)	0.460
GLU, mmol/L	6.22(5.84–6.94)	6.05(5.75–6.88)	6.38(5.99–7.62)	<0.001^b^
Prediabetes	250(75.3%)	139(76.4%)	111(74%)	0.618
Diabetes	82(24.7%)	43(23.6%)	39(26%)	
TBiL, umol/L	16.65(11.82–21.67)	15.50(12.05–21.22)	17.90(11.70–22.75)	0.190
ALT, IU/L	56.50(33.00–110.75)	56.00(32.00–103.00)	58.00(37.00–123.25)	0.515
AST, IU/L	54.00(33.00–90.00)	52.00(32.75–86.25)	54.50(33.00–92.25)	0.435
ALB, g/L	43.80(40.72–46.60)	43.90(41.00–46.80)	43.60(40.25–46.50)	0.472
ALP, IU/L	85.00(69.00–110.00)	82.00(68.00–105.25)	92.00(71.00–122.25)	0.024^b^
GGT, IU/L	41.50(26.00–93.25)	42.50(25.75–83.75)	40.50(25.75–96.25)	0.824
Hb, g/L	141.00(126.00–153.00)	141.00(127.00–154.25)	137.50(125.00–153.00)	0.388
PLTs, 10^9^/L	114.00(77.25–153.25)	118.50(84.50–166.50)	108.50(73.00–142.25)	0.014^b^
WBCs, 10^9^/L	5.24(4.13–6.52)	5.36(4.27–6.59)	4.93(3.91–6.48)	0.118
APRI	1.23(0.65–2.61)	1.05(0.63–2.18)	1.41(0.69–3.04)	0.036^b^
FIB-4	3.43(1.94–6.01)	3.13(1.81–5.21)	3.93(2.30–7.36)	0.003^b^

CHC, chronic hepatitis C; SVR, sustained virologic response; HCV RNA, hepatitis C virus ribonucleic acid; FPG, fasting plasma glucose; TBiL, total bilirubin; ALT, alanine aminotransferase; AST, aspartate aminotransferase; ALB, albumin; ALP, alkaline phosphatase; GGT, gamma glutamyltranspeptidase; Hb, hemoglobin; PLTs, platelets; WBCs, white blood cells; APRI, aspartate aminotransferase to platelet ratio index; FIB-4, Fibrosis-4. ^a^The χ^2^ test or Fisher’s exact probability test was used to examine the categorical variables such as sex, age, genotype and diabetes status, and the Mann-Whitney U test was used to examine the continuous variables between the improved and unimproved group. ^b^Values were statistically significant at P < 0.05.

### Risk factors for unimproved glucose after SVR

Further multivariate analysis was performed to identify the factors related to glucose metabolic improvement in subjects with prediabetes/diabetes who subsequently achieved an SVR. Here we included demographic parameters, liver function-related parameters and other parameters. The results are shown in Table 6. Multivariate analysis revealed baseline characteristics (age ≥ 61 years, HCV-RNA ≥ 6.55 log IU/mL, FPG ≥ 5.84 mmol/L, TBiL ≥ 16.65 μmol/L and ALT ≥ 110.75 IU/L) were independent risk factors for unimproved glucose after SVR. This result implied that older age, higher viral load, and worse liver function all interfered with the improvement in glucose metabolism.

**Table 6:** The independent variables that influence the glucose improvement.

Parameters	Improved	Unimproved	OR (95%CI)	*P - value*^a^
	(N = 182)	(N = 150)		
Sex (Ref-male)	96(52.7%)	77(51.3%)	1	
Female	86(47.3%)	73(48.7%)	0.656(0.325–1.324)	0.239
Age, years (Ref-<44)	54(29.7%)	23(15.3%)	1	
≥44 and <51	40(22%)	38(25.3%)	2.038(0.921–4.510)	0.079
≥51 and <61	47(25.8%)	43(28.7%)	1.792(0.787–4.082)	0.165
≥61	41(22.5%)	46(30.7%)	2.816(1.217–6.513)	0.016^b^
Genotype (Ref-1)	157(86.3%)	129(86%)	1	
2	7(3.8%)	4(2.7%)	1.378(0.408–4.652)	0.605
3	16(8.8%)	11(7.3%)	1.066(0.479–2.375)	0.875
Others and unknown	1(0.5%)	1(0.7%)	0.733(0.431–1.246)	0.251
HCV-RNA, log IU/mL (Ref<5.17)	53(29.1%)	29(19.3%)	1	
≥5.17 and <6.07	39(21.4%)	44(29.3%)	1.848(0.854–3.998)	0.119
≥6.07 and <6.55	44(24.2%)	38(25.3%)	2.005(0.888–4.525)	0.094
≥6.55	46(25.3%)	39(26%)	2.359(1.061–5.247)	0.035^b^
FPG, mmol/L (Ref-<5.84)	60(33%)	23(15.3%)	1	
≥5.84 and <6.22	46(25.3%)	37(24.7%)	2.962(1.358–6.464)	0.006^b^
≥6.22 and <6.94	33(18.1%)	50(33.3%)	4.540(2.074–9.941)	<0.001^b^
≥6.94	43(23.7%)	40(26.7%)	3.325(1.479–7.471)	0.004^b^
TBiL, mmol/L (Ref-<11.82)	43(23.6%)	40(26.7%)	1	
≥11.82 and <16.65	58(31.9%)	25(16.7%)	1.056(0.364–3.064)	0.921
≥16.65 and <21.67	42(23.1%)	41(27.3%)	3.763(1.027–13.794)	0.046^b^
≥21.67	39(21.4%)	44(29.3%)	5.010(1.150–21.832)	0.032^b^
ALT, IU/L (Ref-<33.00)	49(26.9%)	33(22%)	1	
≥33.0 and <56.50	43(23.6%)	41(27.3%)	1.908(0.735–4.954)	0.184
≥56.50 and <110.75	49(26.9%)	34(22.7%)	1.952(0.663–5.747)	0.224
≥110.75	41(22.5%)	42(28%)	4.468(1.126–17.729)	0.033^b^
AST, IU/L (Ref-<33.00)	45(24.7%)	33(22%)	1	
≥33.00 and 54.00	48(26.4%)	39(26%)	0.684(0.259–1.808)	0.444
≥54.00 and 90.00	46(25.3%)	37(24.7%)	0.532(0.164–1.729)	0.294
≥90.00	43(23.6%)	41(27.3%)	0.330(0.067–1.620)	0.172
ALB, g/L (Ref-<40.72)	41(22.5%)	42(28%)	1	
≥40.72 and <43.80	46(25.3%)	34(22.7%)	0.608(0.276–1.339)	0.217
≥43.80 and <46.60	46(25.3%)	38(25.3%)	0.817(0.365–1.828)	0.622
≥46.60	49(26.9%)	36(24%)	0.651(0.288–1.473)	0.303
ALP, IU/L (Ref-<69.00)	48(26.4%)	34(22.7%)	1	
≥69.00 and <85.00	52(28.6%)	30(20%)	0.673(0.308–1.471)	0.321
≥85.00 and <110.00	47(25.8%)	36(24%)	1.084(0.501–2.348)	0.838
≥110.00	35(19.2%)	50(33.3%)	2.104(0.863–5.126)	0.102
GGT, IU/L (Ref<26.00)	45(24.7%)	37(24.7%)	1	
≥26.00 and <41.50	45(24.7%)	39(26%)	0.628(0.286–1.382)	0.248
≥41.50 and <93.25	49(26.9%)	34(22.7%)	0.476(0.196–1.156)	0.101
≥93.25	43(23.6%)	40(26.7%)	0.587(0.211–1.630)	0.306
Hb, g/L (Ref-<26)	42(23.1%)	39(26%)	1	
≥26 and <141	44(24.2%)	40(26.7%)	1.245(0.570–2.720)	0.582
≥141 and <153	46(25.3%)	31(20.7%)	0.761(0.315–1.837)	0.544
≥153	50(27.5%)	40(26.7%)	1.111(0.407–3.033)	0.838
PLTs, 109/L (Ref<77.25)	35(19.2%)	48(32%)	1	
≥77.25 and <114.00	50(27.5%)	32(21.3%)	0.553(0.234–1.310)	0.178
≥114.00 and <153.25	42(23.1%)	42(28%)	0.956(0.346–2.644)	0.931
≥153.25	55(30.2%)	28(18.7%)	0.450(0.143–1.420)	0.173
WBCs, 109/L (Ref-<4.132)	38(20.9%)	45(30%)	1	
≥4.132 and <5.24	48(26.4%)	34(22.7%)	0.487(0.219–1.083)	0.078
≥5.24 and <6.52	49(26.9%)	35(23.3%)	0.440(0.194–0.999)	0.050
≥6.52	47(25.8%)	36(24%)	0.602(0.256–1.413)	0.244
APRI (Ref- ≤ 2)	130(71.4%)	91(60.7%)		
>2	52(28.6%)	59(39.3%)	0.895(0.337–2.379)	0.824
FIB-4 (Ref- ≤ 3.25)	94(51.6)	61(40.7%)		
>3.25	88(48.4)	89(59.3%)	1.219(0.481–3.090)	0.676

OR, odds ratio; CI, confidence interval; HCV RNA, hepatitis C virus ribonucleic acid; FPG, fasting plasma glucose; TBiL, total bilirubin; ALT, alanine aminotransferase; AST, aspartate aminotransferase; ALB, albumin; ALP, alkaline phosphatase; GGT, gamma glutamyltranspeptidase; Hb, hemoglobin; PLTs, platelets; WBCs, white blood cells; APRI, aspartate aminotransferase to platelet ratio index; FIB-4, Fibrosis-4. ^a^Binary logistic regression was performed. ^b^Values were statistically significant at *P* < 0.05.

## Discussion

HCV infection induces glucose metabolic disorder. A previous study reported that the prevalence of diabetes in HCV patients is approximately the age-standardized prevalence (9.7%) of diabetes, while the prevalence of prediabetes in HCV patients is higher than the age-standardized prevalence (15.5%) in the general Chinese population^25^. Our study found different prevalences in the enrolled cohort; a total of 278 (25.5%) and 89 (8.16%) patients were diagnosed with prediabetes and diabetes, respectively. This discrepancy may be attributed to a population selection bias, as our study enrolled patients from only Sichuan Province, a southwest region of China. However, both studies implied that the prevalence of prediabetes was higher in patients with HCV infection.

Whether glucose metabolism improved after the eradication of the virus remains to be elucidated. In our study, we observed that the clearance of HCV induced a significant improvement in glycaemic control in 990 patients who had achieved an SVR, as demonstrated by the reduction in the glucose level in this group. However, we did not find a decrease in glucose levels in the other 100 non-SVR patients. To reduce the variation between the SVR and non-SVR groups, we used PSM to normalize the baseline characteristics and finally enrolled 99 patients in the SVR group and 99 patients in the non-SVR group. The fact that decreases in the FPG level occurred in patients with an SVR but in non-SVR patients further demonstrated that the clearance of HCV could improve glucose metabolism. A number of studies reported similar findings with a decreased rate of glucose abnormalities in CHC patients who achieved an SVR. For instance, Kawaguchi T *et al*. enrolled 89 CHC patients and treated them with PegIFN-α (alone or with ribavirin). The results showed that patients who achieved an SVR had a significant decrease in the homeostasis model assessment of insulin resistance (HOMA-IR) index based on fasting glucose and insulin levels (P < 0.05); additionally, the expression of IRS1/2, two transducers of the insulin signal pathway, in hepatocytes showed a threefold increase,^19^. Concordantly, in the Virahep-C multicentre study, Conjeevaram HS *et al*. demonstrated that patients who achieved an SVR have a significant improvement in the HOMA-IR level compared to genotype 1 CHC patients who did not respond to antiviral treatment or relapsed^21^. Similar data were reported for prediabetes CHC patients, which also demonstrated that the eradication of HCV could improve glucose abnormalities^22^. Together with our findings, these results suggest that the eradication of HCV may be associated with glucose improvements, reducing the risk of prediabetes/T2DM development. However, in contrast to the above findings, in a study of 30 CHC patients from Japan treated with PegIFN-α and ribavirin patients showed no changes in HOMA and glucose levels after 6 months of treatment^23^. Additionally, Brandman *et al*. enrolled 50 noncirrhotic, nondiabetic CHC patients, of whom 23 were treated with PegIFN-α with or without ribavirin and 27 were untreated^24^. These authors reported that insulin resistance did not appear to be strongly associated with SVR and that HCV treatment might improve insulin resistance regardless of the virologic response. This discrepancy might be related to the lower number of patients enrolled in their study. However, these conflicting reports suggest that further investigations of the factors influencing the improvement in plasma glucose after the eradication of this virus are needed.

The condition of patients, such as baseline liver function and baseline glucose levels, may influence glycometabolism improvements. We reported that “age, baseline HCV-RNA, GLU, TBiL and ALT” influenced the improvement in glycaemic control. Among these parameters, “AGE ≥ 61 years, HCV-RNA ≥ 6.55 log IU/mL, FPG ≥ 5.84 mmol/L, TBiL ≥ 16.65 μmol/L and ALT ≥ 110.75 IU/L” were risk factors. Accordingly, a study by Chehadeh W *et al*. showed that older age (≥ 50 years) was a risk factor for T2DM in HCV patients^20^. In our study, we also found that older age (≥ 61 years) (OR: 2.816; 95% CI: 1.217–6.513, P < 0.016) is a risk factor for glucose improvement. Our results showed that higher baseline HCV RNA levels are a negative factor for glucose improvement. Moucari R *et al*. conducted a study that showed that IR was associated with a high serum HCV RNA level^26^. Because a high serum HCV RNA level is associated with IR, we speculate that patients with a higher baseline HCV RNA levels have more difficulty improving their glucose than patients with lower baseline HCV RNA levels. Takashi Oono *et al*. showed that TBiL is an independent parameter contributing to a HOMA-IR of 2.5 or more (OR: 5.396; 95% CI: 1.822–15.978, P = 0.002) in univariate analysis, while there was no significance in multivariate analysis^27^. Our results showed that TBiL was a risk factor for glucose improvement through multivariate analysis but only when it was obviously elevated. This discrepancy might be related to the factors included in the analysis model being different between the study by Takashi Oono *et al*. and our study. However, the main factor may be that an obviously increased TBiL indicates certain damage in liver cells. It also reported the correlation between ALT and glucose improvement. A meta-analysis from Fraser A *et al*. demonstrated that ALT was a risk factor leading to diabetes (OR: 2.02; 95% CI: 1.59–2.58)^28^. Our study showed that elevated ALT was a risk factor for glucose improvement (OR: 4.468, 95% CI: 1.126–17.729), also indicating that liver damage hampers the improvement of glucose metabolism. In 2014, many oral direct-acting antiviral agents (DAAs) were approved for HCV treatment. Compared to interferon and ribavirin therapies alone, DAAs have higher potency, higher safety, lower side effects and shorter treatment durations and have been widely used in most countries. Insulin resistance did not impair the response of CHC patients treated with DAAs^29^. Currently, many studies on DAA treatments for HCV have also demonstrated the potential benefit in glucose metabolism after SVR^30–36^. A retrospective single-center observational study from Philip Weidner *et al*. investigated 281 patients receiving DAA ± Ribavirin and measured FPG. The results showed a significant drop in FPG levels after SVR24 in both the whole cohort and T2DM (n = 28) patients^30^. Another study screened 65 diabetic HCV patients who received sofosbuvir ± ribavirin treatment regimens and showed a statistically significant decline in FPG and HbA1C values at SVR24^31^. A larger sample size of 2,435 diabetic HCV patients was treated with ribavirin-free DAA therapy. That study showed improved glycaemic control in patients, as indicated by decreased mean HbA1C after the eradication of HCV after 3–15 months^32^. Furthermore, in HCV patients with T2DM, Alessia Ciancio *et al*. also conducted a prospective case-control study that enrolled 122 consecutive patients and showed that viral eradication by DAAs reduced fasting glucose and HbA1C; this was not observed in untreated patients after SVR12, and 20.7% of patients could reduce or suspend their antidiabetic therapy^33^. Meanwhile, in patients without T2DM, Luigi E. Adinolfi *et al*. conducted a prospective case-control study that enrolled 133 consecutive HCV patients with advanced liver fibrosis (F3-F4) and showed that viral eradication by DAAs reduced HOMA-IR and serum glucose, while no variation occurred in untreated patients after SVR12^34^. In addition, a recent report from Alessandro Gualerzi *et al*. found similar results, showing that an improvement in glucose metabolism occurred both in diabetes and non-diabetes patients after antiviral treatment by DAAs^35^. As a result, The eradication of HCV may improve glycometabolism, regardless of the treatment regime with PR or DAAs. However, the above studies been limited to relatively short-term follow-up (3–15 months after SVR), and more studies are needed to verify whether these results are maintained over the long term. Our study had some limitations. First, the study was a retrospective design, and the use of a PR treatment regimen was abandoned in many countries. Second, we did not obtain enough important data for HbA1C. Third, the data of mass index (BMI) were not complete in all patients, so we could not analyze this confounding factor in T2DM. Studies showed that higher BMI was an additional risk factor for IR^13,24^. And higher BMI was associated with pre-diabetes in HCV patients^10^. Another study also reported that BMI is predictive of diabetes mellitus in CHC patients^37^. Last, whether the benefits of glucose abnormality improvement are maintained with a longer follow-up period deserves to be studied, and we will discuss this in a future study. Our study had some strengths. First, our study enrolled 1090 patients, which was a large sample size. Second, we conducted a controlled study that included SVR and non-SVR groups and analyzed the pre-treatment and post-treatment parameters. Our findings suggest the idea that the eradication of HCV after treatment may improve glycometabolism. Third, we selected both subjects with prediabetes and diabetes to study the independent variables that may influence glucose improvement.

In conclusion, patients with HCV infection had a higher prevalence of abnormal glycometabolism. It could be improved after viral eradication, indicating that HCV may influence glycometabolism. Moreover, older age, higher baseline HCV RNA, GLU, TBiL and ALT are associated with less glucose improvement, and we should pay more attention to these factors.

## Supplementary information

Supplementary Tables
