# Supplementary material for: Hepatitis C Virus Clearance with Glucose Improvement and Factors Affecting the Glucose Control in Chronic Hepatitis C Patients
Source: Sci Rep. 2020 Feb 6;10:1976. doi: 10.1038/s41598-020-58786-x (PMC7005176; doi:10.1038/s41598-020-58786-x)
Supplement: Supplementary file 1 — Supplementary Tables [file 41598_2020_58786_MOESM1_ESM.docx]

**Hepatitis C Virus Clearance with Glucose Improvement and Factors Affecting the Glucose Control in Chronic Hepatitis C Patients**

Man Yuan^1,2^, Juan Zhou^3^, Lingyao Du^1,2^, Libo Yan^1,2^, Hong Tang*^1,2^

1 Center of Infectious Diseases, West China Hospital of Sichuan University, Chengdu, China

2 Division of Infectious Diseases, State Key Laboratory of Biotherapy and Center of Infectious Diseases, West China Hospital, Sichuan University, Chengdu, China

3 Department of Laboratory Medicine, West China Hospital, Sichuan University, Chengdu, China

**Corresponding author details**

Hong Tang, Center of Infectious Diseases, West China Hospital of Sichuan University, No.37 Guoxue Alley, Chengdu, Sichuan Province, 610041, China.

Phone: +86 28 85422650/ Fax: +86 28 85423052

e-mail: htang6198@hotmail.com

**Supplementary Table S1** The treatment in T2DM CHC patients with and without an SVR

| **Treatment** | **T2DM patients**  **(N=89)** | **SVR(+)**  **(N=82)** | **SVR(-)**  **(N=7)** |
| --- | --- | --- | --- |
| Antidiabetic medications | 35(39.3%) | 33(40.2%) | 2(28.5%) |
| Insulin | 11 | 10 | 1 |
| Hypoglycaemic agents | 24 | 23 | 1 |
| Lifestyle treatment | 54(60.7%) | 49(59.8%) | 5(71.5%) |

T2DM，type 2 diabetes mellitus; CHC, chronic hepatitis C; SVR, sustained virologic response.

**Supplementary Table S2** Comparison of baseline and post-treatment HbA1C in the T2DM CHC patients with and without an SVR

| **Parameters** | **SVR(+) (N=22)** | | ***P*-value^a^** | **SVR(-) (N=4)** | | ***P*-value^a^** |
| --- | --- | --- | --- | --- | --- | --- |
|  | **Pre-treatment** | **Post-treatment** |  | **Pre-treatment** | **Post-treatment** |  |
| HbA1C, % | 6.30(6.15-7.95) | 5.90(5.45-6.25) | 0.002^b^ | 5.85(5.05-5.90) | 5.85(5.10-6.37) | 0.715 |

T2DM，type 2 diabetes mellitus; CHC, chronic hepatitis C; HbA1C, hemoglobin A1C; SVR, sustained virologic response; ^a^The Wilcoxon rank-sum test was used to examine the differences between pre-treatment and post-treatment profiles in SVR (+) and SVR (-) group, respectively. ^b^Values were statistically significant at *P* < 0.05.
